# Individual participant data meta-analyses compared with meta-analyses based on aggregate data

**DOI:** 10.1186/1745-6215-12-S1-A57

**Published:** 2011-12-13

**Authors:** Catrin Tudur Smith, James Oyee, Maura Marcucci, Maroeska Rovers, Alfonso Iorio, Richard Riley, Paula Williamson, Mike Clarke

**Affiliations:** 1 North West Hub for Trials Methodology Research, University of Liverpool, UK; 2 Department of Medicine, University of Perugia, Perugia, Italy; 3 Departments of Epidemiology, Biostatistics & HTA and Operating Rooms, UMC St Radboud, The Netherlands; 4 Department of Clinical Epidemiology and Biostatistics, McMaster University, Hamilton, ON, Canada; 5 Public Health, Epidemiology and Biostatistics, University of Birmingham, UK; 6 All-Ireland Hub for Trials Methodology Research, Queen’s University Belfast, UK

## Background

Meta-analysis based on individual participant data (IPD) is widely accepted as the most reliable approach and has been described as the ‘gold standard‘ for systematic reviews. An IPD approach often allows more powerful, consistent and thorough analyses but does require additional resources compared with meta-analyses based on aggregate data (AD). Several empirical comparisons of IPD with AD meta-analyses have been published, some of which show that IPD meta-analyses can differ in important ways from meta-analyses based on AD. For example, the importance of including as much follow-up as possible on all randomised participants and data from all relevant trials was shown in separate empirical comparisons [1-3] whilst Duchateau [4] found substantial differences between IPD and literature-based AD meta-analyses, mainly due to different approaches to analysis. An unpublished review [5] summarised results from across 25 studies, showing that for two thirds of the comparisons AD estimated effect sizes with less precision and tended to overestimate the IPD effect but differences were small in most comparisons.

## Objectives

We have undertaken a Cochrane systematic review of empirical studies that compared IPD and AD meta-analysis to explore key reasons for the differences.

## Methods

The Cochrane Methodology Register, CENTRAL, MEDLINE and EMBASE were searched using a predefined set of search terms. Studies that report an empirical comparison of IPD meta-analysis against AD meta-analysis of randomised trials were assessed for inclusion, by two reviewers independently. Data were extracted by two reviewers independently and stored in a central database.

## Results

Over forty empirical studies have satisfied the inclusion criteria for this review. Estimates of effect size and precision obtained from IPD and AD will be compared and differences will be discussed. Results will help inform the ongoing debate about whether, and when IPD may be most valuable.
